# Association of vitamin D levels with anthropometric and adiposity indicators across all age groups: a systematic review of epidemiologic studies

**DOI:** 10.1530/EC-23-0394

**Published:** 2024-01-04

**Authors:** Behnaz Abiri, Majid Valizadeh, Amirhossein Ramezani Ahmadi, Shirin Amini, Mohammad Nikoohemmat, Faeze Abbaspour, Farhad Hosseinpanah

**Affiliations:** 1 Obesity Research Center, Research Institute for Endocrine Sciences, Shahid Beheshti University of Medical Sciences, Tehran, Iran; 2 Isfahan Endocrine and Metabolism Research Center, Isfahan University of Medical Sciences, Isfahan, Iran; 3 Department of Nutrition, Shoushtar Faculty of Medical Sciences, Shoushtar, Iran

**Keywords:** vitamin D, 25(OH)D, anthropometric, adiposity

## Abstract

**Objectives:**

It has not been established whether vitamin D deficiency is associated with anthropometric state; therefore, this systematic review examined the relationship between serum vitamin D levels with anthropometrics and adiposity across different ages.

**Methods:**

Studies that examined vitamin D deficiency with adiposity measures in different age groups were searched in the PubMed, Scopus, Embase, and Google Scholar databases until November 2023. Two investigators independently reviewed titles and abstracts, examined full-text articles, extracted data, and rated the quality in accordance with the Newcastle–Ottawa criteria.

**Results:**

Seventy-two studies, with a total of 59,430 subjects, were included. Of these studies, 27 cross-sectional studies and one longitudinal study (with 25,615 participants) evaluated the possible link between 25(OH)D serum concentrations and anthropometric/adiposity indices in the pediatric population. Forty-two cross-sectional studies and two cohort investigations (with 33,815 participants) investigated the relationship between serum 25(OH)D levels and adiposity measures in adults and/or the elderly population. There is evidence supporting links between vitamin D deficiency and obesity, and revealed an inverse association between vitamin D and adiposity indicators, specifically in female subjects. However, the effects of several confounding factors should also be considered.

**Conclusion:**

Most published studies, most of which were cross-sectional, reported a negative association between vitamin D and female adiposity indicators. Therefore, serum vitamin D levels should be monitored in overweight/obese individuals.

## Introduction

Obesity results from excess fat accumulation and a positive energy balance, contributing to various chronic diseases and reduced life expectancy (1). Approximately 650 million adults, roughly 13% of the global adult population, were overweight or obese, with 340 million children and adolescents aged 5–19 years classified as overweight or obese in 2016 (2).

Micronutrient deficiencies, notably hypovitaminosis D, are common in obese patients (3, 4). Extensive observational studies like NHANES III and Framingham have linked obesity to an increased risk of hypovitaminosis D (5, 6). Vitamin D serves various functions, including maintaining calcium homeostasis and bone health, while also influencing metabolic processes, immunity, cellular proliferation, and differentiation, among other effects such as anti-inflammatory, antiatherogenic, cardioprotective, and neuroprotective impacts (7, 8). A global prevalence of widespread vitamin D deficiency has been identified, with deficiency rates rising by 13%, insufficiency rates reaching 40%, and notably higher rates observed in Asian countries (9, 10).

Obesity is commonly linked with reduced vitamin D levels regardless of various factors such as age, gender, season, study region, or smoking status (11). The coexistence of obesity and hypovitaminosis D represents a dual public health concern globally, prompting the need for investigating the underlying pathophysiology of this relationship. Mechanisms contributing to low vitamin D levels in obesity involve volumetric dilution, sequestration into adipose tissue, limited sunlight exposure, and reduced vitamin D synthesis in adipose tissue and the liver (11). Studies have suggested that low vitamin D levels may influence adipose tissue differentiation and growth, impacting obesity through gene expression regulation or by modulating parathyroid hormone (PTH), calcium, and leptin (11, 12, 13). While several observational studies have explored the link between vitamin D status and body weight, comprehensive evaluations of the relationship between serum vitamin D levels and anthropometric and adiposity indicators in both adults and children are lacking.

Hence, in this groundbreaking systematic review, we significantly contribute to the existing literature by taking a comprehensive and inclusive approach to evaluate the intricate relationship between serum 25(OH)D levels and adiposity. Unlike prior studies that primarily focused on specific age groups or relied on limited adiposity measures such as body mass index (BMI) and waist circumference (WC), our research spans diverse age groups and considers a broader set of indicators, including BMI, WC, HC (hip circumference), WHR (waist-to-hip ratio), and body fat mass percentage. Our findings reveal intriguing patterns across the life span, adding a valuable dimension to the understanding of vitamin D deficiency in the context of obesity. This holistic evaluation provides a nuanced perspective on the association between serum 25(OH)D levels and various aspects of adiposity, offering a more comprehensive overview compared to previous reviews. This research is important as it seeks to fill the existing gap in knowledge concerning the intricate association between vitamin D levels and obesity. By conducting a systematic review, the study intends to shed light on the underlying mechanisms and implications of this relationship, thereby contributing to the development of effective public health strategies.

## Methods

We conducted a systematic review of studies that assessed the relationship between serum vitamin D levels with anthropometric and adiposity indices in children, adolescents, adults, and the elderly. Serum 25(OH)D was used as a proxy measure for vitamin D levels.

### Search strategy

The PubMed, Scopus, Embase, and Google Scholar databases were used to identify relevant publications. Two authors (BA and SA) independently searched papers published until November 2023 using (‘25-hydroxy vitamin D’ OR ‘vitamin D’ OR ‘cholecalciferol’ OR ‘25(OH)D’) AND (‘BMI’ OR ‘body mass index’ OR ‘weight’ OR ‘obese’ OR ‘obesity’ OR ‘waist’ OR ‘waist circumference’ OR ‘adiposity’ OR ‘adipose’, OR ‘fat’) as keywords. No restrictions were imposed on publication time or language. The reference lists of relevant articles were also reviewed by the authors to determine whether any publications were missing. All of the studies included in this systematic review were published in English. Data extraction was done independently by two investigators (BA and MN). In the event of any disagreement, three authors (BA, MN, and FH) discussed it among them to resolve the disagreement. Owing to the differences in the comparisons of the included studies (differences in exposures, outcomes, participants, and settings), diversity of applied statistical tools in the comparisons of the included studies, and lack of data that could be pooled, we performed a qualitative systematic review. The systematic review was conducted following the Preferred Reporting Items for Systematic Reviews and Meta-Analyses (PRISMA 2020) Statement (14).

Table 1 shows the PICOS (population, intervention/exposure, comparator, outcome, and setting) items used to conduct the systematic review. Owing to the methodological approach, no ethical approval was required.

**Table 1:** PICOS (population, intervention/exposure, comparator, outcome, and setting) criteria used to perform the systematic review.

PICOS	Criteria
Population	Healthy general population across all age groups
Intervention/exposure	Serum 25(OH)D
Comparator	Statistical tools (OR, HR, RR)
Outcome	Anthropometric/adiposity indices
Setting	Observational studies

OR, odds ratios; HR, hazard ratios; RR, relative risk.

### Eligibility criteria

Publications with abstracts that suggested vitamin D levels were investigated in relation to anthropometric and adiposity variables were reviewed in full. Studies met the inclusion criteria if they: i) had observational design; (ii) were carried out in apparently healthy individuals (without chronic diseases, such as diabetes, liver diseases, cancer, or chronic kidney disease); and (iii) used serum 25(OH)D levels as a proxy for vitamin D state. However, clinical trials, reviews, editorials, and studies on nonhuman models, were excluded. Sex and age ranges were not strictly defined in this systematic review.

### Study selection

Each title and abstract collected during the initial search was independently evaluated by two authors after removing duplicates. To ensure that eligibility and exclusion criteria were met, the two authors assessed full-text articles. The researchers consulted each other whenever they disagreed.

### Data extraction and quality assessment

The following information was recorded in a data mining sheet: first author, publication year, country, ethnicity, design of the study, sample size, sex of participants, age, study population, method of 25(OH)D measurements, cut offs for vitamin D status, anthropometric indices investigated in the study and their cut off points, adjustments, and main findings. We assessed the quality of observational studies using the Newcastle–Ottawa Scale (NOS) (15).

## Results

### Literature search and study selection process

**Figure 1:**
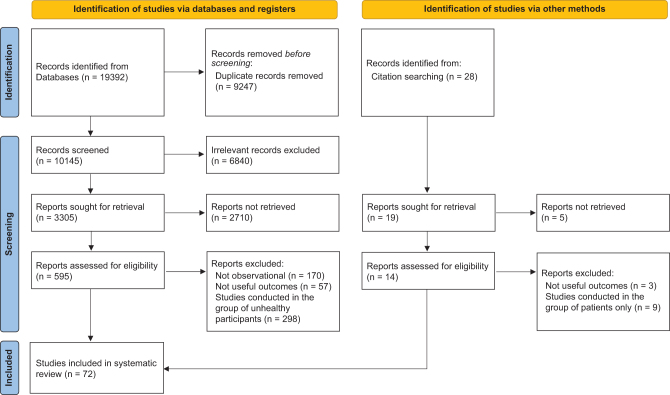
PRISMA flow diagram for the selection process of the studies.

From databases, 19392 studies were initially found. Following the removal of 9247 duplicate articles, 7342 were excluded after scanning the titles/abstracts as they had no relevance to the present systematic review. After careful screening of 2803 full texts, we also excluded 2731 more studies because they evaluated the relationship between serum levels of 25(OH)D with an outcome other than anthropometric/adiposity indices, were clinical trials, animal or *in vitro* studies in design, editorial, and reviews, or the participants of the studies were unhealthy (with chronic diseases, such as diabetes, cancer, or chronic kidney disease). Ultimately, 72 studies (12, 13, 16, 17, 18, 19, 20, 21, 22, 23, 24, 25, 26, 27, 28, 29, 30, 31, 32, 33, 34, 35, 36, 37, 38, 39, 40, 41, 42, 43, 44, 45, 46, 47, 48, 49, 50, 51, 52, 53, 54, 55, 56, 57, 58, 59, 60, 61, 62, 63, 64, 65, 66, 67, 68, 69, 70, 71, 72, 73, 74, 75, 76, 77, 78, 79, 80, 81, 82, 83, 84, 85) with 59,430 participants in total, published between 1981 and 2023, could be considered for the systematic review. Figure 1 illustrates the flowchart for selecting studies.

#### Characteristics of the studies conducted in children/adolescent population

The current systematic review identified 27 cross-sectional studies (16, 17, 18, 19, 20, 21, 22, 23, 24, 25, 26, 27, 28, 29, 30, 31, 32, 33, 34, 35, 36, 37, 38, 39, 40, 41, 42) and one longitudinal study (43) that assessed the potential relationship between serum 25(OH)D levels and anthropometric/adiposity indices in children. The studies about the relation between 25(OH)D serum level and anthropometric indices in pediatric were carried out in the following countries: four studies from Turkey, three from Spain, two from Denmark, five from the USA, two from Brazil, four from Italy and eight others from Iran, Korea, China, Thailand, England, Saudi Arabia, Sri Lanka, and Germany. The included studies were conducted between 2008 and 2021. Tgncluded studies had samples ranging from 51 to 12292 in this age group. Participant ages ranged from 3 months to 21 years.

All included studies involved both sexes, except three investigations (26, 39, 40) that were conducted in females (39, 40) and males (26) only. The included studies mostly considered the following potential confounders: sex, age, weight, BMI, cohort characteristics, Tanner stage, fat mass index, body fat mass percentage, BMI *Z*-score, parental education, pubertal breast stage, physical activity, dietary or supplemental vitamin D and calcium intake, race, ethnicity, season of blood collection, and height measures.

The characteristics of the articles are shown in Table 2.

**Table 2:** Characteristics of the studies investigating the association between vitamin D and/or PTH with anthropometric indices in children/adolescents.

First author (reference number), year	Country, ethnicity	Study design	Sample size	Sex	Age of participants (years)	Study population	Method of 25(OH)D measurement	Cutoffs for vitamin D status	Anthropometric indices investigated in the study and their cutoff points	Adjustments	Main findings	Study quality (NOS)
Küçükali (19), 2021	Turkey; NM	Cross sectional	162 (84/78)	F/M	12–18	84 obese children and 78 healthy children	25(OH)D: Liquid chromatography–tandem mass spectrometry with a commercial kit. PTH: colorimetric method using ready-made kits	Normal: 25(OH)D >20 ng/mL and PTH <65 pg/mL.	Obesity: BMI >95th percentile. Normal: 15th–85th percentiles.	–	Obese children had higher level of PTH. Bioavailable and free vitamin D were lower in the obese group. There was no difference in terms of total vitamin D between groups.	4
Durá-Travé (18), 2020	Spain; Caucasian	Cross-sectional	630 (282/348)	F/M	7.2–15.2	282 sever obese participants and 348 healthy control children	Chemiluminescence immunoassay/two-site chemiluminescent enzyme-labeled immunometric assay in an Immulite analyzer	Normal: 25(OH)D ≥30 ng/mL, Insufficiency: 20–29 ng/mL, deficiency: <20 ng/mL. Hyperparathyroidism: PTH levels >65 pg/mL.	Severe obesity: BMI *Z*-score >3.0, 99th percentile). Control: *Z*-score between −1.0 (15th percentile) and +1.0 (85th percentile).	–	Suboptimal vitamin D status and high levels of PTH are a common feature in pediatric with severe obesity.	5
Geserick (43), 2020	Germany; NM	Longitudinal	2733 (317/2416)	F/M	3 months–17 years	LIFE child study population	Electrochemiluminescence assays	Electrochemiluminescence assays	Weight and height.	–	Obese children had lower SDS for 25(OH)D and higher SDS for PTH than the control cohort.	7
Gün (17), 2020	Turkey; –	Cross-sectional	150 (92/58)	F/M	5–17	92 obese children and 58 healthy controls without chronic diseases	25(OH)D was measured using commercial kit	Severely lacking: 25(OH)D <5 ng/mL, <15 ng/mL as lacking, 15−20 ng/mL as deficiency.	Normal weight at BMI ≤85th percentile, overweight at 85th–95th percentile, and obese at ≥95th percentile.	–	The prevalence of vitamin D deficiency was higher in obese children compared to normal-weight and overweight children.	4
Durá-Travé (20), 2020	Spain; Caucasian	Cross-sectional	236 adolescents with severe obesity (BMI z-score > 3.0, 99th percentile)	F/M	10.2–15.8	Adolescents with severe obesity	25(OH)D was measured by a high-specific chemiluminescence immunoassay/and PTH assessed by a highly specific solid-phase, two-site chemiluminescent enzyme-labeled immunometric assay	Normal: 25(OH)D ≥30 ng/mL, Insufficiency: 20–29 ng/mL, deficiency: < 20 ng/mL.	BMI, body composition, WC, WHR.	–	Subjects with vitamin D deficiency had significantly elevated values for BMI *Z*-score, WC, waist *Z*-score, body fat percentage, fat mass index, and PTH than those with normal vitamin D status.	5
Adikaram (21), 2019	Sri Lanka; –	Cross-sectional	202	F/M	5–15	Children with BMI >2 SDS above the median for age and sex based on WHO	Immunoassays	Deficiency: 25(OH)D <20 ng/mL, Insufficiency: 25(OH)D: 20 –29 ng/mL. PTH >65 pg/mL.	Above +2 SDS for WC of relevant references and percentage fat mass >28.6 in boys and >33.7% in girls were considered as high.	–	Vitamin D deficiency was significantly high in children with obesity and showed negative correlations with indicators of adiposity.	5
Plesner (22), 2017	Denmark; North European White	Cross-sectional	3627 (1484/2143)	F/M	6–18	Children and adolescents with overweight/obesity and 2143 population-based controls	Electrochemiluminescence binding assay/ Electrochemiluminescent immunoassay	Normal: 25(OH)D ≥50 nmol/L), insufficiency: <50 nmol ≥ 30 nmol/L, Deficiency:<30 nmol/L.	Obese: BMI-SDS ≥2.33 or above the 99th percentile, overweight: BMI-SDS ≥1.28 < 2.33), lean: BMI-SDS <1.28 or below the 90^th^ percentile.	–	Vitamin D deficiency was common among children and adolescents with obesity. The degree of obesity was independently associated with lower serum 25(OH)D concentrations.	7
Giudici (23), 2017	Brazil; NM	Cross-sectional	198	F/M	14–18	Adolescents who were participated in the Health Survey–Sao Paulo study	High-performance liquid chromatography	Deficiency: 25(OH)D < 20 ng/mL, Insufficiency: 25(OH)D: 20 –29 ng/mL.	Weight, WC, hip circumference were measured. Weight status was determined according to BMI-for-age growth charts. Normal weight: between the 3rd and 85th percentiles; overweight: the 85th and 97th percentiles; obese: above 97th percentile.	–	25(OH)D was lower in overweight and obese adolescents.	4
Alemzadeh (24), 2016	USA; Caucasian, Hispanic, African American	Cross-sectional	152	F/M	13.2–17.8	Obese adolescents (BMI >95th percentile for age)	Nichols RIA/Nichols immunochemiluminometric assay	Normal: 25(OH)D ≥75 nM), insufficiency: 50–74.9 nM, Deficiency:<50 nmol/L.	BMI >95th percentile for age, body composition.	–	Hypovitaminosis D and vitamin D–deficient groups had higher BMI, fat mass, and iPTH, than vitamin D-sufficient group. Fat mass was negatively correlated with 25(OH)D (*r* = −0.40, *P* < 0.0001), it was positively correlated with iPTH (*r* = 0.46, *P* < 0.0001).	4
Saber (25), 2015	Saudi Arabia; NM	Cross-sectional	96 (60/36)	F/M	3–13	Healthy, overweight and obese children	Chemiluminescence immunoassay technology	–	Underweight, normal weight and overweight were defined as BMI below the 10th, between 10th and 85th and above the 85th percentile, respectively, according to WHO standards.	–	In obese children PTH level was significantly higher and 25(OH)D was lower than controls. 25(OH)D was negatively correlated with PTH and BMI. PTH was not positively correlated with BMI percentile.	4
Di Nisio (26), 2015	Italy; NM	Cross-sectional	108	M	11–14	Boys were recruited in the Operative Unit of Medicine, District of Salerno, section of Sapri (SA, Italy)	Competitive chemiluminescent immunoassay/sandwich-type chemiluminescent immunoassay	Normal: 25(OH)D ≥30 ng/mL, Insufficiency: 20–29 ng/mL, deficiency: ≤ 20 ng/mL.	Normal weight, overweight, and severe obesity defined as CDC-BMI <85^th^ percentile and ≥85th percentile, and ≥95^th^ percentile.	Weight, cohort characteristics, Tanner stage	Lean and overweight groups had similar mean 25(OH)D levels and were below the sufficiency threshold. A considerable fraction of subjects featured by 25(OH)D insufficiency or deficiency was observed in both normal weight and overweight–obese groups.	4
Petersen (27), 2015	Denmark; Danish	Cross-sectional	782	F/M	8–11	Date from the Optimal well-being, development and health for Danish children	Automated chemiluminescent immunoassay/chemiluminescent immunoassay on ADVIA Centaur XP	Vitamin D deficiency and insufficiency defined as serum 25(OH)D concentration <25 and ≤50 nmol/L, respectively.	Height, weight, BMI, WC, body composition.	FMI, BMI *Z*-score, parental education	Serum 25(OH)D was negatively associated with BMI *Z*-scores and FMI. The association with BMI became nonsignificant after adjustment. But the negative association with FMI remained after adjustment.	5
Reesukumal (28), 2015	Thailand; Thai	Cross-sectional	159	F/M	6–12	Healthy children	Electrochemiluminescence immunoassay on Elecsys 2010 analyzers	Sufficiency: 25(OH)D level ≥75 nmol/L; hypovitaminosis D: 25(OH)D level <75 nmol/L; insufficiency: 25(OH)D level 50–74.9 nmol/L; vitamin D deficiency: 25(OH)D level <50 nmol/L.	BMI percentile groups (< 85th vs ≥ 85th percentile).	–	Children with hypovitaminosis D had a higher mean BMI percentile than the vitamin D-sufficient group. PTH levels in the children with hypovitaminosis D were significantly higher than in the children with normal levels of vitamin D.	4
Rusconi (29), 2015	Italy; Caucasia, Africans, Asians, others	Cross-sectional	120	F/M	10.2 ± 2.8	Obese children with different values of vitamin D (25(OH)D <20 ng/mL (group Ι) and 25(OH)D >20 ng/mL (group ΙΙ))	–	25(OH)D <20 ng/mL (group Ι) and 25(OH)D > 20 ng/mL (group ΙΙ).	Weight, height, body composition.	–	The two groups were similar for BMI SDS and fat mass SDS, while showed differences for PTH.	4
Vierucci (30), 2014	Italy; Italian	Cross-sectional	427	F/M	10–21	Healthy adolescents	Radioimmunoassay/immunoradiometric assay	Vitamin D deficiency < 20; insufficiency 20–30 ng/mL; sufficiency ≥ 30 ng/mL. PTH ≥65.0 ng/L defined as hyperparathyroidism.	Weight status was categorized in normal, overweight and obese according to criteria for subjects <18 years and according to the WHO for subjects aged 18–21 years.	–	Increased risk of hypovitaminosis D in overweight-obese adolescents compared to subjects with normal BMI, was shown. 25(OH)D levels were inversely related to PTH and BMI-SDS.	5
Chung (31), 2014	Korea; Korean	Cross-sectional	1212	F/M	4–15	Children who visited Bundang CHA Medical Center for checkup of their health and growth status	Chemiluminescence immunoassay/electrochemiluminescence immunoassay	Deficiency: 25(OH)D, <20 ng/mL; insufficiency: 25(OH)D, 20–30 ng/mL, and sufficiency 25(OH)D, ≥30 ng/mL.	Normal weight defined by BMI 3rd to 84th percentile; overweight was defined by BMI ≥85th percentile for age and sex based on Korean standard growth curve.	–	The level of 25(OH)D was significantly lower in overweight group than in normal weight group. The PTH levels were significantly higher in vitamin D deficient group compared to vitamin D insufficiency and sufficiency group.	6
Vierucci (32), 2013	Italy; Italian	Cross-sectional	652	F/M	Children (2.0–10.9 years) and adolescents (11.0–21.0 years)	Healthy children and adolescents	Radioimmunoassay/immunoradiometric assay	Vitamin D deficiency < 20; insufficiency 20–30 ng/mL; sufficiency ≥30 ng/mL, PTH ≥65.0 ng/L defined as hyperparathyroidism.	Weight status was categorized in normal, overweight and obese according to criteria for subjects <18 years and according to the WHO for subjects aged 18–21 years.	–	Significant increased risk of hypovitaminosis D in overweight and obese subjects compared to individuals with normal BMI was observed. But weight status did not affect PTH levels.	5
Oliveira (33), 2013	Brazil; Brazilian	Cross-sectional	160 (83/77)	F/M	15–17	Healthy adolescents	RIA kit/chemiluminescence immunoassay	Vitamin D deficiency and insufficiency were ≤10 ng/mL and between 10–30 ng/mL, respectively. The intact PTH level between 15 and 65 pg/mL being considered normal.	Weight, height, WC, %BF were measured. Nutritional status was assessed by BMI according the WHO recommendations. Excessive weight was defined as BMI above 1 SD. Abdominal adiposity was estimated using cutoff points corresponding to the 90th centiles.	–	Serum 25(OH)D levels were statistically lower in adolescents with weight excess, abdominal obesity, and higher levels of PTH.	4
Turer (37), 2013	USA; White, African American, Latino, others	Cross-sectional	12292	F/M	6–18	Children who were enrolled in the 2003–2006 National Health and Nutrition Examination Survey	Radioimmunoassay	Deficiency: <20 ng/mL, insufficiency: 20–30 ng/mL	Height, weight, BMI.	Race, age, sex, season, TV use, PA, vitamin D, and milk intake	Vitamin D deficiency is highly prevalent in overweight and obese children.	7
Alemzadeh (35), 2012	USA; Caucasian, Hispanic, African American	Cross-sectional	133	F/M	13.1–17.9	Obese adolescents	Nichols RIA/Nichols immunochemiluminometric assay	–	Age-adjusted BMI ≥95th percentile.	–	Fat mass was negatively correlated with 25(OH)D, but was positively correlated with PTH.	4
Buyukinan (36), 2012	Turkey; Turkish	Cross-sectional	106	F/M	8–16	Children and adolescents with BMI ≥95th percentile	Electrochemiluminescence	Normal: 25(OH)D ≥30 ng/mL, insufficiency: 20–30, deficiency: < 20 ng/mL. PTH >65 pg/mL was hyperparathyroidism.	Height, weight, BMI, WC. Obesity was defined as BMI ≥ 95th.	–	No significant difference was found between the groups with different vitamin D levels in terms of weight SDS, height SDS, BMI SDS, and WC SDS.	4
Codoñer-Franch (34), 2012	Spain; Caucasian	Cross-sectional	105 (66/39)	F/M	7–14	Obese children	Electrochemiluminescence immunoassays	Vitamin D sufficient: >20 ng/m) or insufficient <20 ng/mL.	Weight, height, WC, and body composition were measured. Obese defined ad SDS-BMI ≥ 2), extremely obese: SDS-BMI >4.	Sex, age, and Tanner stage	Obese children had a significantly lower 25(OH)D and a higher iPTH than nonobese children. Insufficient serum levels of 25(OH)D were detected in 5% of normal children and in 30% of the obese children.	4
Razzaghy-Azar (38), 2010	Iran; NM	Cross-sectional	313	F/M	8–18	Healthy children and adolescents who came to a routine growth monitoring clinic of the university hospital	Radioimmunoassay/immunoradiometric	Severely deficient <12.5 nmol/L; deficient ≥12.5 and <25 nmol/L; insufficient ≥25 and <50 nmol/L; sufficient, ≥50 nmol/L and ≤250 nmol/L and toxic >250 nmol/L.	Height, weight, BMI.	–	The level of 25(OH)D had a negative correlation with BMI-SDS and height-SDS in females, but these correlations were not significant in males.	5
Foo (39), 2009	China; Chinese	Cross-sectional	323	F	15.0 ± 0.4	Apparently healthy adolescent girls	I-radioimmunoassay/immunometric assay	–	Height, weight, BMI, body composition.	Pubertal breast stage, physical activity, dietary calcium and vitamin D intake	A significant positive correlation was found between lean body mass and plasma 25(OH)D. No significant correlation was found between the %BF and vitamin D status.	5
Ashraf (40), 2009	England; African-American	Cross-sectional	51	F	14 ± 2	Obese adolescent girls	Liquid chromatography–tandem mass spectrometry (LC-MS/MS)/two-site immunoradiometric assay	Vitamin D deficiency cutoff: <20 ng/mL	Height, weight, BMI. Obesity was defined as BMI ≥95th.	BMI	It was shown a trend toward a significant negative relationship between 25(OH)D and BMI, PTH. Level of PTH was significantly associated with BMI.	4
Lenders (41), 2009	USA; Black or African American, Hispanic, Summer, North of Atlanta	Cross-sectional	58	F/M	13.0–17.9	Healthy obese adolescents	In-house competitive protein binding assay/enzyme immunoassay	Vitamin D deficiency was defined as 25(OH)D concentration <20 ng/mL.	Height, weight, BMI, body composition.	Age, sex, race, season, vitamin D intake, height measures, Tanner stage	25(OH)D decreased by 0.46 ± 0.22 ng/mL per 1% increment in body fat mass, whereas PTH decreased by 0.78 ± 0.29 pg/mL per 1% increment in visceral adipose tissue.	4
Alemzadeh (42), 2008	USA; Caucasian, Hispanic, African American	Cross-sectional	127	F/M	6.0–17.9	Obese children and adolescents	Nichols radioimmunoassay/Nichols immunochemiluminometric assay	Hypovitaminosis D, 25(OH)D <75 nmol/L; sufficiency, 25(OH)D ≥75 nmol/L; deficiency, 25(OH)D level <50 nmol/L; insufficiency, 25(OH)D of 50–74.9 nmol/L.	Height, weight, BMI, body composition. Obesity was defined as BMI > 95th percentile for age.	Age, sex, ethnicity, season	Hypovitaminosis D and vitamin D–deficient groups had higher BMI, fat mass, and iPTH, than vitamin D-sufficient group. Fat mass was negatively correlated with 25(OH)D, it was positively correlated with iPTH.	4
Çizmecioğlu (16), 2008	Turkey; Turkish	Cross-sectional	301	F/M	11–19	Secondary and high school children	Competitive protein binding assay/chemiluminescence with an Immulite One analyzer	Deficiency: 25(OH)D <10 ng/mL; insufficiency as levels of 25(OH)D between 10 and 20 ng/mL, and a normal vitamin D level as >20 ng/mL.	Height, weight, BMI.	–	It was shown a negative correlation between serum vitamin D level and BMI in obese and overweight subjects whose vitamin D level was below 20 ng/mL.	4

BMI, body mass index; F/M, female/male; FMI, fat mass index; iPTH, intact parathyroid hormone; NOS, Newcastle–Ottawa Scale; PA, physical activity; PTH, parathyroid hormone; SDS, standard deviation score; WC, waist circumference; WHO, World Health Organization.

#### Characteristics of the studies conducted in adult and/or elderly population

Our systematic search identified 42 cross-sectional studies (12, 13, 44, 45, 46, 47, 48, 49, 50, 51, 52, 53, 54, 55, 56, 57, 58, 59, 61, 62, 63, 64, 65, 66, 67, 68, 69, 70, 71, 72, 73, 74, 75, 76, 77, 79, 83, 84, 85) and two cohort investigations (60, 78) (with 33,815 participants) that investigated the potential relationship between 25(OH)D serum concentrations and anthropometric/adiposity indices in adults and/or elderly population. The studies about the link between 25(OH)D serum level and anthropometric indices in adults and/or elderly population were carried out in the following countries: 9 studies from USA, 3 from UK, 3 from Spain, 2 from Turkey, 4 from Saudi Arabia, 2 from Iran, 2 from India, 2 from Italy, 2 from Germany, and 18 others from China, UAE, Pakistan, Australia, Finland, England, Portugal, Malaysia, Bulgaria, Austria, Canada, Denmark, Greece, New Zealand, and the Netherlands. The included studies were conducted between 1981 and 2023. The included studies had samples ranging from 26 to 3113 in this age group. The age of participants was >18 years old. Among the included studies, 27 articles (44, 45, 46, 47, 48, 49, 50, 51, 52, 53, 54, 55, 56, 57, 58, 59, 60, 61, 62, 63, 64, 65, 66, 67, 68, 84, 85) involved both sexes, and 17 investigations conducted in females (12, 13, 72, 73, 74, 75, 76, 77, 78, 79, 80, 81, 82, 83) and males (69, 70, 71) only. The included studies mostly considered the following potential confounders: age, sex, season of blood sampling, smoking, vitamin D status, body fat mess, weight, height, BMI, IGF-1, PTH, UVB, alcohol, tobacco, sports, use of multivitamin supplements, menopausal status/HRT use, physical activity, socioeconomic status, income, job, obesity, education level, lifestyle, sun exposure, lean mass, nutrient intake, residence type, race, ethnicity, serum calcium, magnesium and phosphate, GFR, 25(OH)D, CRP, WC, month of blood collection, marital status, district, area, latitude, phosphorus, serum alanine aminotransferase, creatinine, and IL-6.

The characteristics of the articles are shown in Table 3.

**Table 3:** Characteristics of the studies investigating the association between vitamin D and/or PTH with anthropometric indices in adult and/or elderly population.

First author (reference number), year	Country, ethnicity	Study design	Sample size	Sex	Age of participants (years)	Study population	Method of 25(OH)D measurement	Cutoffs for vitamin D status	Anthropometric indices investigated in the study and their cutoff points	Adjustments	Main findings	Study quality (NOS)
Tejada‑Romero (12), 2022	Spain; NM	Cross-sectional	679 (493/186)	F	60.6 ± 13.6	Healthy postmenopausal women	Immunochemical luminescence/immunochemical luminescence	–	Weight, height, BMI. Obesity was defined as a BMI ≥30.	Age	Obese women had lower levels of 25(OH)D and higher PTH than nonobese.	6
Shan (13), 2022	China; Chinese	Cross-sectional	1505	F	30.04 (23.98–37.81)	Women of childbearing age	Liquid chromatography–tandem mass spectroscopy/electrochemiluminescence immunoassay	–	Height, weight, WC, BMI.	Season, district, area, latitude, age, BMI, PTH, P, ALT, CRE, IL-6, hs-CRP	Insufficient 25(OH)D was significantly related to the risk of elevated WC after adjusting for confounders.	7
Gariballa (48), 2022	UAE; NM	Cross-sectional	648	F/M	≥18	Community free living adults	Electrochemiluminescence	Deficiency:< 20 ng/mL; insufficiency: 20–32 ng/mL; sufficient: >32 ng/mL	Height, weight, BMI.	–	There was no difference in BMI between groups, but, it was higher in vitamin D deficient subjects aged ≥50 and females <50 years.	6
Saleem (72), 2021	Pakistan; NM	Cross-sectional	397 (264/133)	F	20–50	Healthy women with normal fasting glucose	Radioimmunoassay/spectrophotometer	Deficient: serum vitamin D <12 ng/mL; sufficient: vitamin D >30 ng/mL	Weight, height, BMI, WC, and HC. Obesity was defined as BMI ≥30 kg/m^2^.	–	PTH levels were negatively correlated, though nonsignificantly with vitamin D. The mean BMI, WC, HC, WHR, and PTH were not significantly different in vitamin D-deficient as compared to nondeficient obese women.	4
Djafari (49), 2021	Iran; NM	Cross-sectional	178	F/M	60–83	Elderly individuals	Enzymatic method using commercial kits	–	Weight, height, BMI, WC, HC, and fat mass.	Age, sex, income, PA, job, smoking, vitamin D and calcium supplement use	25(OH)D was higher in the highest category of BAI compared to the lowest one. An inverse association between BAI with 25(OH)D was reported. No significant association between the %BF with PTH and 25(OH)D was observed.	4
Menon (69), 2021	India; Indian	Cross-sectional	224	M	20.74 ± 1.43	Healthy military training	Chemiluminescence analyzer	Sufficient: vitamin D ≥30 ng/mL; insufficient: 20–30 ng/mL; deficient: ≤20 ng/mL	Height, weight, BMI, body composition.	–	There was no correlation of 25(OH) cholecalciferol with BMI or FMI.	4
Yaylali (73), 2021	Turkey; NM	Cross-sectional	126 (73/53)	F	17–55	Healthy premenopausal women	High performance liquid chromatography/electrochemiluminescence immunoassay	–	Height, weight, WC, BMI, body composition. Obesity was defined as BMI >30 kg/m^2^.	–	A positive association was observed between PTH and visceral fat. Vitamin D levels were inversely associated with visceral fat.	4
Sharma (74), 2020	Australia; NM	Cross-sectional	76 (50/26)	F	61.9–68.5	Healthy normal and overweight postmenopausal women	Chemiluminescent immunoassays	–	Weight, height, BMI, WC, HC, and body composition. Normal:BMI <25 kg/m^2^, and overweight: BMI >27 kg/m^2^.	–	Women in the highest visceral adipose tissue quartile had significantly lower 25(OH)D.	4
Albassam (75), 2019	Saudi Arabia; NM	Cross-sectional	265 (179/86)	F	18–70	Middle-aged Saudi women	Electrochemiluminescence immunoassay/Milliplex MAP Human Bone Magnetic Bead Panel	–	Weight, height, BMI, WC, HC, NC, body composition.	Age and menopausal status	NC was inversely associated with 25(OH)D and PTH. In the nonobese, WHR was inversely associated with PTH.	5
Saarnio (50), 2018	Finland; Caucasian	Cross-sectional	595	F/M	37–47	Healthy men and women	IDS enzyme immunoassay kit/immunoluminescence-based method by Immulite1000	–	Weight, height, BMI.	Age, physical activity, smoking	In women, 25(OH)D levels did not differ among the BMI groups. In men, 25(OH)D was lower in obese men than in normal-weight. Altogether, obese subjects had lower 25(OH)D than normal-weight. In women, PTH was significantly higher in obese compared to normal weight Altogether, the difference was significant.	5
Kord‑Varkaneh (51), 2018	Iran; NM	Cross-sectional	178	F/M	60–83	Elderly subjects	Enzymatic method	–	Weight, height, BMI, WC.	Age, sex, PA, smoking, marital, supplement use	No significant correlation between 25(OH)D with BMI and WC.	4
Raposo (52), 2017	Portugal; NM	Cross-sectional	500	F/M	53 (41–67)	Adults registered in primary health-care centers	Chemiluminescent immunoassay/electrochemiluminescent immunoassay	Deficiency <12 ng/mL; inadequacy ≥12 and <20 ng/mL; sufficiency ≥20 ng/mL	Weight, height, WC, HC. Participants were classified into: underweight (BMI <18.5 kg/m^2^), normal range (≥18.5 to <25), pre-obese (≥25 to <30 kg/m^2^) and obese (≥30 kg/m^2^) categories.	Age and sex	The serum 25(OH)D levels were negatively associated with BMI. Positive associations between PTH with BMI and WC were found.	5
Trevisan (76), 2017	Italy, Caucasian	Cross-sectional	218	F	≥65	Healthy older women	Radioimmunoassay/two-step immunoradiometric assay	25(OH)D level <50 nmol/L was considered inadequate; PTH value >60 pg/mL defined hyperparathyroidism	Weight, height, BMI, body composition.	Age, 25(OH)D, PTH, month of blood collection	Fat mass showed a significant negative association with 25(OH)D. Binary logistic analysis revealed a protective effect of adiposity on secondary hyperparathyroidism.	4
Walsh (53), 2016	UK, Caucasian	Cross-sectional	233	F/M	25–40 and 55–75	Healthy men and women in different weight groups	Immunoassay/direct measurement by immunoassay	NM	Weight, height, BMI.	Age, sex	Serum 25(OH)D was inversely correlated with BMI. PTH did not differ by BMI group.	4
Shafinaz (54), 2016	Malaysia; Malays, Chinese, Indians, others	Cross-sectional	858	F/M	<30 to >50	Permanent teachers who worked in the selected government secondary schools	Electro chemiluminescence immunoassay	Serum 25(OH)D <20 ng/mL was considered as vitamin D deficient	Weight, height, BMI, WC, body composition. Underweight: BMI <17.5 kg/m^2^; normal weight: 17.5–22.9 kg/m^2^; overweight: BMI 23.0–27.9 kg/m^2^; obese: BMI ≥ 28.0 kg/m^2^.	Age, sex, ethnicity, sun avoidance score, SBP, BMI, WC, %BF	A significant negative association between serum 25(OH)D with BMI and %BF was observed. Higher BMI and larger WC were significantly associated with lower serum 25(OH)D level.	6
Al-Daghri (55), 2015	Saudi Arabia; NM	Cross-sectional	830	F/M	18–50	Apparently healthy individuals	ELISA	Sufficient vitamin D >50 nmol/L, insufficient: 25–50 nmol/L and deficient: <25 nmol/L	Weight, height, BMI, WC, HC, arm circumference.	Age, BMI, glucose, cholesterol, TG, LDL, HDL	Vitamin D insufficiency was significantly associated with abdominal obesity in males.	6
Wright (56), 2015	India; Caucasian, Black, Asian, Hispanic	Cross-sectional	336	F/M	35–65	Middle-aged overweight and obese healthy adults	Radioimmunoassays	–	Weight, height, BMI, WC, HC, body composition.	Age, sex, race, season, supplement use, PTH	Total and central adiposity but not peripheral adiposity predicted low plasma 25(OH)D.	4
George (57), 2015	USA; Caucasian	Cross-sectional	714	F/M	18–65	Healthy adults	High-performance liquid chromatography (HPLC) kit/chemiluminescent assay	–	Weight, height, BMI, WC, body composition.	Age, sex, height, ethnicity, serum calcium, magnesium and phosphate, GFR, smoking	25(OH)D was not associated significantly with BMI and WC.	6
Tosunbayraktar (58), 2015	Turkey; NM	Cross-sectional	90	F/M	18–63	Healthy individuals with various BMIs	–	Deficiency: 25(OH)D <20 ng/mL; and ≥20 ng/mL as sufficiency	Weight, height, BMI, WC, HC, body composition.	–	Overweight and obese groups had higher PTH and lower 25(OH)D levels than normal weight group. The overweight group had higher 25(OH)D and lower PTH than obese group.	4
Sorkin (77), 2014	USA; white and black	Cross-sectional	239	F	46–78	Sedentary postmenopausal women without diabetes	RIA/immunoradiometric assay	–	–	Race, age, and time	25(OH)D was inversely related to visceral abdominal fat, %BF, and PTH.	4
Shinkov (59), 2014	Bulgaria; NM	Cross-sectional	1952	F/M	F: 49.5 ± 14.2 M: 47.2 ± 14.1	Healthy adult population group	Liquid chromatography–tandem mass spectrometry/chemiluminescence	Sufficient vitamin D >50 nmol/L, insufficient: 25–50 nmol/L and deficient: <25 nmol/L	Weight, height, BMI.	Sex, age, education, residence type, BMI and smoking	25(OH)D levels were significantly lower in obese females than in the normal weight females. In the males, the 25(OH)D levels did not differ among the BMI groups. The increase in BMI by 1 kg/m^2^ was associated with an increase in the prevalence of vitamin D deficiency in the young females by 1%.	7
Shapses (78), 2013	USA; NM	Retrospective	383	F	24–75	Premenopausal women with regular menstruating and postmenopausal women were at least 2 years since their last menstruation	RIA	–	Weight, height, BMI, body composition.	–	Higher serum levels of PTH and lower 25(OH)D in the obese subjects compared to leaner subjects were observed.	5
González-Molero (60), 2013	Spain; NM	Prospective	961	F/M	18–68	Participants in Pizarra cohort study	Electrochemiluminescence	Deficiency: 25(OH)D ≤20 ng/mL	Weight, height, BMI, WC, HC.	Age, sex, season, iPTH, and presence of diabetes	Neither obesity at baseline nor the development of obesity were significantly associated with vitamin D status. In nonobese subjects 25(OH)D ≤17 ng/mL was associated with an increased risk of developing obesity in the next 4 years.	5
Jungert (61), 2012	Germany; NM	Cross-sectional	131	F/M	66–96	Independently living participants of the longitudinal study on nutrition and health status.	Direct electrochemiluminescence immunoassay	25(OH)D <25.0 nmol/L as deficient, 25.0–49.9 nmol/L as insufficient and ≥50.0 nmol/L as adequate	Weight, height, BMI, WC, HC, body composition.	Age, lifestyle, iPTH	25(OH)D3 was inversely associated with BMI, HC, and BF in women. Total BF was a negative predictor of 25(OH)D3 in women even after controlling for confounders, whereas the associations between BMI, HC, and 25(OH)D3 lost statistical significance after adjusting for iPTH.	4
Ardawi (70), 2012	Saudi Arabia; NM	Cross-sectional	834	M	20–74	Men were recruited at random during a health survey from 40 primary health-care centers	Competitive chemiluminescence immunoassays/electrochemiluminescent assay	Deficiency: 25(OH)D <50 nmol/L	Weight, height, BMI, WC, HC. BMI ≥25–<30 kg/m^2^), and an obese group (BMI ≥30 kg/m^2^).	Age, obesity, education, season, lifestyle, sun exposure	Vitamin D deficiency was common among older and obese men with no education and sedentary lifestyle sampled during summer and spring.	6
Puntus (62), 2011	Austria; NM	Cross-sectional	1009	F/M	25–76	Healthy adults	Enzyme-based protein binding assay/immunoradiometric assay	–	Weight, height, BMI. BMI 18.5–25 kg/m^2^ (normal), BMI 25–30 kg/m^2^ (overweight), BMI >30 kg/m^2^ (obese).	Age	Increasing BMI was associated with a significant fall of 25(OH)D in pre- and postmenopausal women, but with a significant rise in PTH in women before menopause.	7
Sukumar (79), 2011	USA; Caucasian	Cross-sectional	211	F	25–71	Normal weight and overweight/obese women	Radioimmunoassay	–	Weight, height, BMI. Body composition. Normal-weight: BMI <25, overweight and obese-class I: BMI 25–35, obese-class II–III: BMI >35.	Lean mass, PA, nutrient intake	Higher BMI was associated with greater levels of PTH, but lower 25(OH)D.	4
Ardawi (80), 2011	Saudi Arabia; NM	Cross-sectional	1172	F	50.9 ± 12.6	Women were recruited at random during a health survey from 40 primary health-care centers	Direct competitive chemiluminescence immunoassays/direct sandwich chemiluminescence immunoassays	25(OH)D ≤75 nmol/L as deficiency/insufficiency; >75 nmol/L as sufficient; hyperparathyroidism: iPTH >7.0 pmol/L	Weight, height, BMI, WC, HC. BMI ≥25–<30 kg/m^2^), and an obese group (BMI ≥30 kg/m^2^).	Age, obesity, education, season, lifestyle, sun exposure	Serum 25(OH)D was lower and intact PTH higher in the upper quintiles of BMI and WHR.	7
Hayek (63), 2011	Canada; NM	Cross-sectional	2168	F/M	>18 years	Inuit adults participated in the International Polar Year Inuit Health Survey	Chemiluminesent assays	Serum 25(OH)D was compared to different cutoffs: >75, >50 nmol/L, 75–37.5 nmol/L, and <37.5 nmol/L	Weight, height, BMI, WC, Body composition. For men, a healthy WC was defined as <102 cm and for women <88 cm.	–	Healthy WC was a significant predictor of better 25(OH)D level in adults.	7
Frost (71), 2010	Denmark; Caucasian	Cross-sectional	783	M	20–29	Young men	Radioimmunoassay/nonimmunoflourometric assay	Deficiency: 25(OH)D <50 nm; insufficiency 25(OH)D <75 nm.	Weight, height, BMI, WHR, Body composition.	Age, alcohol, tobacco, sports, use of multivitamin supplements, and season, BMI	An inverse relationship between 25(OH)D and BMI was found in participants with a high BMI only, and increase in BMI of 1 kg/m^2^ corresponded to a decrease in 25(OH)D of 1.7 nm. PTH was inversely associated with BFM in vitamin D-insufficient subjects.	6
Valiña-Tóth (64), 2010	USA; African American	Cross-sectional	98	F/M	≥35 years	Healthy, overweight, adult	Liquid chromatography–tandem spectrometry/electrochemiluminescence method	Deficiency: 25(OH)D ≤50 nmol/L; insufficiency: 51-74 nmol/L; optimal: ≥75 nmol/l	Weight, height, BMI, WHR, Body composition.	Age, sex and seasons	PTH was directly correlated with total, truncal and extremity FM, while 25(OH)D was related inversely to truncal FM.	4
Muscogiuri (65), 2010	Italy; NM	Cross-sectional	39	F/M	41.4 ± 12.4	Subjects with no known history of diabetes mellitus	Chemiluminescence immunoassay radioimmunoassay/ enzyme chemiluminescence immunoassay	–	Weight, height, BMI.	–	There was a correlation between 25(OH)D and BMI (*r* = −0.58; *P* = 0.01), and PTH (*r* = −0.44; *P* < 0.01). BMI was the most powerful predictor of 25(OH)D level.	4
Moschonis (81), 2009	Greece; NM	Cross-sectional	112	F	60.3 ± 5.0	Postmenopausal healthy women	Chemiluminescence immunoassays	–	Weight, height, BMI, WC, Body composition.	Age, UVB, PTH, and IGF-1, PA	No significant associations were observed between anthropometric indices of body mass and serum 25(OH)D levels	4
Rueda (66), 2008	Spain; NM	Cross-sectional	298	F/M	42.9±10.6	Severely obese patients	Radioimmunoassay/chemiluminescence immunoassay	Insufficient: 25(OH)D < 20 pg/mL; PTH reference range: 10–65 pg/mL	Weight, height, BMI, Body composition.	Age, sex, BMI, %FM, season	Insufficient 25(OH)D was associated with higher BMI. Elevated PTH levels were linked to higher BMI and higher FM percentage.	4
Macdonald (82), 2008	UK; Caucasian	Cross-sectional from Aberdeen Prospective Osteoporosis Screening Study	3113	F	54.8 (2.3)	Postmenopausal women	HPLC	25(OH)D ≥28 ng/mL with those <28 ng/mL	Weight, height, BMI.	Age, weight, height, menopausal status/HRT use, physical activity and socioeconomic status	25(OH)D was lower and PTH higher in the top quintile of BMI.	7
Bolland (83), 2006	New Zealand; NM	Cross-sectional	116	F	62.6 ± 5.9	Healthy community-dwelling postmenopausal women	Radioimmunoassay/Allegro assay	Vitamin D insufficiency; 25(OH)D <50 nmol/L	Weight, height, BMI, body composition.	Vitamin D insufficiency, body fat mess	PTH was positively correlated with weight, regional and total fat mass, and %BF, and negatively correlated with 25(OH)D. On multivariate analysis, PTH was positively related to %BF. For 25(OH)D, there were negative correlations with PTH, total fat mass, trunk fat, and pelvic fat. On multivariate analysis, 25(OH)D was negatively related to pelvic fat mass.	4
Snijder (67), 2005	Netherlands; NM	Cross-sectional	453	F/M	≥66	Older men and women	Competitive binding protein assay/immunoradiometric assay	Deficiency: 25(OH)D <10 ng/mL; insufficiency: < 20 ng/mL	Weight, height, BMI, WC, WHR, body composition.	Age, season, smoking, sex	Higher BMI, WC, and BF were associated with lower 25(OH)D and with higher PTH.	5
Parikh (68), 2004	USA; Caucasian, African-American, other	Cross-sectional	302 (154/148)	F/M	Obese: 37.6 ± 9.4; nonobese: 36.6 ± 11.4	Healthy adults	Competitive binding assay/ two-site immunoenzymometric assay	NM	Weight, height, BMI, body composition.	NM	Serum PTH was positively correlated with both BMI and BF. 25(OH)D was negatively correlated with BMI and BF.	5
Ford (44), 2005	USA; white, African American, Mexican American	Cross-sectional	8421	F/M	≥20	Noninstitutionalized civilian U.S. population	Radioimmunoassay	Deficiency: 25(OH)D < 25 ng/mL	Abdominal obesity was defined as WC ≥102 cm for males and ≥88 cm for females.	Age, sex, smoking, serum factors, PA	Inverse association was present for quintiles of 25(OH)D levels and abdominal adiposity.	7
Reis (45), 2007	USA, Southern California; NM	Cross-sectional	1070	F/M	44–96	All adults living in the southern California community of Rancho Bernardo	CLIA	NM	Weight, height, BMI, WC.	Age, season, and major lifestyle factors	There was no significant association between 25(OH)D and abdominal obesity.	4
Bell (46), 1985	USA; white	Cross-sectional	26 (12/14)	F/M	20–35	Normal white subjects	Competitive protein binding assay/ radioimmunoassay	NM	Weight, height, BMI.	NM	PTH was higher and 25(OH)D was lower in the obese than in the nonobese individuals.	4
Compston (47), 1981	England, NM	Cross-sectional	42 (22/20)	F/M	Obese: 22–49; nonobese: 19–52	Obese patients before intestinal bypass r gastric partitioning versus healthy normal controls	Competitive protein-binding assay	NM	Weight, height, BMI.	NM	The mean plasma 25(OH)D level was significantly lower in the obese group than in age-matched controls.	4
Vilaca (84), 2022	UK, NM	Cross-sectional	200	F/M	25–40 or 55–75	Community-dwelling men and women from South Yorkshire	Autoanalyzers	NM	Weight, height, BMI, body composition.	Age, sex	Compared with normal weight, obese individuals had lower 25(OH)D.	5
Avila Castillo (85), 2023	Germany, Caucasian	Cross-sectional	1032 (533/499)	F/M	40–79	Adult population of LIFE-Adult-Study	Chemiluminescent enzyme immunometric assay	Deficiency: 25(OH)D <20 ng/mL	BMI, WC, HC, WHR, %BF.	NM	Low levels of 25(OH)D are linked to higher BMI.	7

%BF, percentage of body fat; ALT, alanine aminotransferase; BAI, body adiposity index; BMI, body mass index; CRE, creatinine; F/M, female/male; FMI, fat mass index; HC, hip circumference; GFR, glomerular filtration rate; hs-CRP, high-sensitivity C-reactive protein; IGF-1, insulin-like growth factor 1; iPTH, intact parathyroid hormone; NC, neck circumference; NM, not mentioned; NOS, Newcastle–Ottawa Scale;P, phosphorus; PA, physical activity; PTH, Parathyroid hormone; SDS, standard deviation score; WC, waist circumference; WHR, waist-to-hip ratio.

#### Serum 25(OH)D and anthropometric measurements

Different methods were used to assess serum vitamin D concentrations, including chemiluminescent immunoassay (CLIA), electrochemiluminescence immunoassay (ECLIA), radioimmunoassay (RIA), enzyme-linked immune sorbent assay (ELISA), immunoassay (IA), enzyme IA (EIA), protein-binding assay (PBA), chromatography, although three articles did not report the using method.

Anthropometric status was assessed based on weight, BMI, WC, WHR, and body composition.

### The relationship between vitamin D with anthropometric and adiposity indicators in children/adolescents population

In a study by Durá-Travé (18), among 282 participants with severe obesity and 348 healthy control children group, vitamin D deficiency was more frequent (*P* < 0.05) in the obesity group (44.5 vs 11.5% and 22.4 vs 3.9%, respectively). In Geserick *et al.*’s study (43) obese children had significantly lower 25(OH)D SDS (standard deviation score) levels (−0.43) than the reference group. According to the Codoñer-Franch *et al.* (34) obese children had a significantly lower 25(OH)D level (*P* = 0.002) compared with nonobese children. The obese group had significantly higher vitamin D deficiency rates compared to the control group in another study (*P* < 0.001) (17). Furthermore, a study (30) demonstrated an increased risk of hypovitaminosis D in overweight/obese adolescents (OR 3.89) compared to those with a normal BMI. Serum 25(OH)D levels were inversely related to BMI-SDS (*r* = −0.141, *P* = 0.007).

According to a cross-sectional study by Saber *et al.* (25) obese children had significantly lower 25(OH)D levels (21.02 vs 29.45 ng/mL) than controls. There was a negative correlation between serum 25(OH)D with PTH, weight, and BMI (*r* = −0.45, −0.55, −0.47, *P* < 0.01, respectively).

A cross-sectional research by Lenders *et al.* on 58 healthy obese adolescents (41) found 25(OH)D decreased by 0.46 ± 0.22 ng/mL per 1% increment in body fat mass (*P* = 0.05).

The cross-sectional study by Alemzadeh *et al.* (42) among 127 subjects aged 13.0 ± 3.0 years, found that hypovitaminosis D and vitamin D-deficient subjects had higher BMI, fat mass, and iPTH compared to vitamin D-sufficient subjects (*P* < 0.01). Also, fat mass was negatively correlated with 25(OH)D (*r* = −0.40, *P* < 0.0001) regardless of seasonal and racial/ethnic factors. In other works, by the same research group (24, 35) between obese adolescents, a negative correlation was found between fat mass and 25(OH)D (*P* < 0.001).

As reported by Chung *et al.* (31), the level of 25(OH)D in overweight children was significantly lower than that of normal weight children (17.1 ± 5.1 ng/mL vs 19.1 ± 6.1 ng/mL, *P* < 0.001). There was an independent association between overweight and vitamin D deficiency (OR 2.21; 95% CI 1.62–3.01).

According to Ashraf *et al.* (40), there is a trend toward a significant relationship between 25(OH)D with BMI (*r* = −0.24; *P* = 0.087) and PTH (*r* = −0.24; *P* = 0.088).

According to Buyukinan *et al.*’s study (36), children and adolescents with a BMI ≥95th percentile did not show any significant differences in terms of weight, height, BMI, and waist circumference between the three groups with deficient, insufficient, and normal vitamin D levels.

There was also a cross-sectional study, by Durá-Travé *et al.* (20), showing that adolescents with severe obesity who had vitamin D deficiency had significantly (*P* < 0.05) elevated BMI *Z*-scores, waist *Z*-scores, body fat percentages, fat mass indexes, and PTH values than those with normal vitamin D state. The serum 25(OH)D levels were negatively correlated (*P* < 0.05) with body fat percentage, fat mass index, and PTH.

Lean and overweight boys (based on CDC-BMI percentiles) had similar mean 25(OH)D levels (*P* = 0.160) and were below sufficiency thresholds according to an analysis by Di Nisio *et al.* (2015) (26). However, there was a large proportion of subjects with insufficient or deficient 25(OH)D in both groups (normal weight: 45/59, 76 %; overweight/obese: 45/49, 92%, *P* = 0.03).

In the study by Foo *et al.* (39), in adolescent girls, lean body mass and plasma 25(OH)D levels were significantly correlated (*r* = 0.446; *P =* 0.001). However, the correlation between body fat percentage and vitamin D status was not significant (*r* = 0.104; *P* > 0.05). According to Giudici *et al.* (23), compared to normal-weight participants, those with overweight had a lower 25(OH)D. All measures of BMI, weight, and WC were negatively associated with 25(OH)D (*P* < 0.05). Other researchers (16) found that serum vitamin D levels were negatively correlated with BMI in overweight/obese subjects with vitamin D levels <20 ng/mL (*r* = −0.186, *P* < 0.01).

Bioavailable and free vitamin D were lower in the obese group. However, the total vitamin D level between the two groups did not differ, according to Küçükali *et al.* (19). In another study by Turer *et al.*, vitamin D deficiency is highly prevalent in overweight and obese children. (37). In a study by Oliveira *et al.* (33) serum 25(OH)D levels were statistically lower in adolescents with excess weight, abdominal obesity, and a high level of PTH (*P* < 0.05).

Another cross-sectional study by Petersen *et al.* (27) showed a negative relationship between serum 25(OH)D and BMI *Z*-scores and fat mass index (*P* = 0.001). However, the association with BMI *Z*-scores became nonsignificant when the model was adjusted for parental education (−0.03, 95% CI −0.07, −0.001, *P* = 0.14).

Plesner *et al.* (22) found that 16.5% of obese children and adolescents showed vitamin D deficiency, with an OR 3.41 (CI 2.27–5.71; *P* < 0.0001) in comparison with peers with normal weight. An increase in risk of hypovitaminosis D was observed in overweight (OR 5.02) and obese (OR 5.36) subjects compared to normal weight subjects (32).

There was a difference in BMI percentile between children with hypovitaminosis D and children who have sufficient vitamin D (56.7 ± 33.9 vs 42.6 ± 36.0; *P* = 0.04) according to a cross-sectional study (28). According to a multivariate analysis, high BMI percentile and high PTH levels were the parameters related to 25(OH)D concentration <75 nmol/L.

Another evaluation (21) among children with obesity could not observe any significant associations between vitamin D deficiency and the anthropometric or metabolic derangements.

In the study by Rusconi *et al.* (29), 59 had 25(OH)D <20 ng/mL (group Ι) and 61 had 25(OH)D > 20 ng/mL (group ΙΙ) were recruited. The two groups were similar for BMI SDS and fat mass SDS.

Razzaghy-azar *et al.* (38) reported that 25(OH)D level had a negative correlation with BMI-SDS and height-SDS in girls (*P* = 0.01 and 0.039, respectively), but these correlations were not significant in boys.

### The relationship between vitamin D with anthropometric and adiposity indicators in adult/elderly population

Albassam *et al.* (75) found a negative correlation (*R* = −0.17; *P* < 0.05) between neck circumference, a surrogate for upper subcutaneous fat, and 25(OH)D in both obese and nonobese participants.

Gonzalez-Molero *et al.* (60) found that neither obesity at baseline (OR 0.98, 95% CI 0.69–1.40, *P* = 0.93) nor obesity after undergoing a second evaluation (OR 0.80, 95% CI 0.48–1.33, *P* = 0.39) was significantly related to vitamin D status. Yaylali *et al.* (73) demonstrated that vitamin D levels were inversely associated with visceral fat (*P* = 0.002, *r* = −0.366). It has also been shown (12) that obese women have lower 25(OH)D values than nonobese women.

In another analysis by Jungert *et al.* (61), BMI, hip circumference, and body fat were negatively correlated with 25(OH)D, but not in men. Using multiple regression analyses, total body fat was shown to be a negative predictor of 25(OH)D concentrations in women even after adjusting for confounders (*β* = −0.247; *P* = 0.016), whereas after adjusting for iPTH, there was no statistically significant association between BMI, hip circumference, and 25(OH)D. In men, 25(OH)D was not influenced by anthropometric or body composition variables.

The overweight and obese groups in Tosunbayraktar *et al.*’s study (58) had lower 25(OH)D levels when compared to the normal BMI group (*P* = 0.01). Participants in the overweight group had higher levels of 25(OH)D than those in the obese group (*P* < 0.05).

A study by Ardawi *et al.* (80) showed that serum 25(OH)D was lower (*P* = 0.001) in upper quintiles of BMI and WHR. Another cross-sectional study (70) found vitamin D deficiency was prevalent among older and obese men with no education and sedentary lifestyles. Among obese, euglycemic women, 90 (40.7%) were deficient in vitamin D, according to a study (72). There were no significant differences in mean age, BMI, WC, hip circumference, WHR, and PTH between vitamin D-deficient and nondeficient obese women.

Based on Bolland *et al.*’s findings (83) 25(OH)D was negatively correlated with total fat mass, trunk fat, and pelvic fat. On multivariate analysis, 25(OH)D was negatively related to pelvic fat mass (*P* = 0.014; partial *r* ^2^ = 0.05). According to Compston *et al.* (1981) (47) obese individuals had significantly lower plasma 25(OH)D levels than age-matched controls.

In another study by Djafari *et al.* (49), among elderly persons, 25(OH)D (*P* = 0.030) was higher in the highest category of body adiposity index (BAI) compared to the lowest one. Additionally, linear regression demonstrated a negative association between BAI with 25(OH)D (*β* = −0.039, *P* = 0.029).

Healthier WC was also associated with better 25(OH)D concentrations among adults (63). Other studies found no significant correlation between 25(OH)D with WC (45, 51) and BMI (51). According to Macdonald *et al.* (82), women in the top quintile of BMI had lower 25(OH)D (*P* < 0.01). There was a significant correlation between 25(OH)D and BMI (*r* = 0.58; *P* = 0.01). In addition, BMI was highly predictive of 25(OH)D level (*r* = −0.52; *P* < 0.01).

According to another study (69), 25(OH)D is not correlated with BMI or fat mass index in healthy male adults. No significant association was found between anthropometric indices and serum 25(OH)D levels in Moschonis *et al.*'s study (2009) (81) on nonosteoporotic, postmenopausal women.

Among women with different BMI levels, Saarnio *et al.* (50) found no difference in 25(OH)D levels. It was found that 25(OH)D was lower in obese man as compared to normal weight man (48.0 ± 2.4 nmol/L vs 56.4 ± 2.0 nmol/L, *P* = 0.003), but there was no difference between normal weight and overweight group or overweight and obese group. When obesity was examined in both sexes, obese subjects had lower 25(OH)D than normal-weight subjects (50.7 ± 1.6 vs 57.0 ± 1.0 nmol/L, respectively; *P* = 0.003). A significant difference was also found between overweight and obese groups (*P* = 0.023). It was found by Walsh *et al.* (53) that serum 25(OH)D was inversely related to BMI.

A study among male participants, by Frost *et al.* (71) showed that 25(OH)D levels were lower in those with a high BMI; those with a BMI over 25 kg/m^2^ had a lower 25(OH)D (61.4 (27.8) nm vs 66.7 (27.5) nm, *P* = 0.015). In participants with a BMI >25 kg/m^2^, an increase in BMI of 1 kg/m^2^ led to a decrease in 25(OH)D of 1.7 nm (95% CI: −2.8; −0.6, *P* = 0.002), whereas in participants with a BMI <25 kg/m^2^, BMI and 25(OH)D were unrelated (coef: 0.7 (95% CI: −0.6; −0.2)). Body fat mass was inversely related to PTH only in individuals with vitamin D insufficiency. Further, 25(OH)D were associated with lean body mass in adjusted analyses and in participants with low vitamin D levels.

An analysis by George *et al.* (57) showed 25(OH)D was not correlated with BMI (*P* = 0.38) and WC (*P* = 0.99). In the Sharma *et al.* study (74), women with a higher visceral adipose tissue quartile had significantly lower 25(OH)D levels (*P* = 0.05).

A negative correlation was found between serum 25(OH)D with BMI (*r* = −0.4; *P* < 0.0001) and body fat mass (*r* = −0.41; P < 0.0001) among healthy adults (68).

Analysis of the subjects aged ≥65 years, in the Longitudinal Aging Study Amsterdam (67), revealed that after adjusting for potential confounders, higher BMI, WC, and sum of skin folds were statistically significantly related to lower 25(OH)D (standardized *β* values were −0.136, −0.137, and −0.140, respectively; all *P* < 0.05) and with higher PTH (0.166, 0.113, and 0.114, respectively; all *P* < 0.05). In comparison to anthropometric measurements, total body fat percentage had a stronger relationship with 25(OH)D (−0.261).

A secondary analysis of data from middle-aged healthy adults with excess weight (56) indicated that total and abdominal adiposity, but not peripheral adiposity, predicted low plasma 25(OH)D total fat mass index (FMI): *P* = 0.018; android FMI: *P* = 0.052; gynoid FMI: *P* = 0.15; appendicular FMI: *P* = 0.07). An additional retrospective analysis (78) on women found lower levels of 25(OH)D among obese individuals (*P* < 0.001).

Bell *et al.* (46), in their study among 12 obese and 14 nonobese White subjects, demonstrated that mean serum 25(OH)D (8 ± 1 vs 20 ± 2 ng/mL, *P* < 0.001) was significantly lower in the obese than in the nonobese subjects.

There was a significant inverse association between abdominal obesity and the quintiles of 25(OH)D levels in another cross-sectional study (44) involving both sexes.

Valiña-Tóth *et al.* (64) showed in a study of healthy overweight adults that 25(OH)D inversely related to truncal fat mass (*P* = 0.02). Another study (79), among women with BMI 18–57 kg/m^2^, showed that higher BMI was related with lower 25(OH)D levels (*r* > −0.27, *P* < 0.001).

Serum 25(OH)D was found to be inversely related to visceral abdominal fat and percentage fat in a cross-sectional study of overweight and obesity (77).

After adjusting for the confounders, Shan *et al.* (13) found that low 25(OH)D levels were significantly related to elevated WC among women (OR = 1.612 (1.014–2.561). According to Puntus *et al.* (62) increasing BMI significantly reduced 25(OH)D levels in pre- and postmenopausal women (*P* < 0.001 and *P* < 0.05, respectively).

Further research (76) revealed a significant negative relationship between fat mass and 25(OH)D among fit older women (*β* = −3.76, *P* < 0.001).

Researchers examined a total of 830 healthy adults (55) and found that vitamin D insufficiency was significantly linked to abdominal obesity in males (OR 2.75 (CI: 1.1–7.1); P < 0.05).

Shinkov *et al.* (59) reported that 25(OH)D levels were significantly lower in obese females than in the normal weight females (34.6 ± 16.2 vs 38.2 ± 17.8 nmol/L, *P* = 0.014), but, in the males, the 25(OH)D levels did not differ among the BMI groups.

A significant negative correlation was reported by Shafinaz *et al.* (54) between serum 25(OH)D level and body fat percentage (*β* = −0.14). Multivariate linear regression analysis found that higher BMI and larger WC were significantly associated with lower serum 25(OH)D levels (*P* 0.05).

A study by Gariballa *et al.* (48) found that, although BMI did not differ statistically significantly between groups, it was higher among vitamin D deficient older subjects and women <50 years, respectively, compared to individuals with adequate vitamin D or optimal concentrations (*P* = 0.05).

Another study (52) found a negative correlation between serum 25(OH)D levels and BMI (*β*: −0.150; 95% CI: −2.262, −0.037). A study (66) in severely obese subjects found that insufficient 25(OH)D corresponded to higher BMI (insufficient: 47.2 ± 5.6 vs not insufficient: 45.9 ± 4.7 kg/m^2^; *P* = 0.047).

Another cross-sectional investigation (84) among community-dwelling men and women reported that compared with normal weight, obese individuals had lower 25(OH)D levels (*P* < 0.05).

In 2023, Avila Castillo *et al.* (85), in their study on the 1032 adult population of the LIFE-Adult-Study, concluded that low levels of 25(OH)D were linked to higher BMI, while fat mass areas showed a negative correlation with 25(OH)D concentrations only in women.

## Discussion

The present systematic review investigated the correlation between serum 25(OH)D levels and anthropometric and adiposity measurements in healthy individuals of various ages. Some previous meta-analyses included studies that reported BMI (86) and WC (87) as an index of weight status in adults only, while our systematic review has considered different adiposity measures in both adults and children. The results of most of the included studies, but not all papers, showed lower 25(OH)D levels and a higher prevalence of vitamin D insufficiency and deficiency in subjects with higher weight, BMI, WC, WHR, and fat mass percentage, in all age groups. In fact, 25(OH)D levels were inversely associated with BMI, WC, HC, WHR, and body fat mass percentage, specifically in female subjects.

Adiposity indicators were inversely associated with vitamin D status in numerous studies but not in all studies. The association between low vitamin D levels and obesity may be attributed to several factors. First, individuals with obesity might face challenges in acquiring sufficient sun exposure owing to limited mobility or specific clothing choices (88, 89, 90). Second, vitamin D is stored in fat compartments and adipose tissues, particularly in the abdominal area, making its release less accessible in obese individuals (88, 91). Third, individuals with obesity often have an increased demand for vitamin D to support their body weight, but the bioavailability of 25(OH)D is reduced, making it challenging to meet these elevated requirements (88, 91). Fourth, as the concentrations of active vitamin D metabolites increase, they initiate a negative feedback control over hepatic 25(OH)D synthesis. This feedback mechanism led to a reduction in serum 25(OH)D levels, providing an additional explanation for the observed association (91). It is important to note that the full acceptance of these mechanisms is still under investigation (92). Conversely, reducing fat mass has been shown to elevate 25(OH)D levels by releasing stored vitamin D into circulation. In a systematic review, Mallard *et al.* (93) concluded that weight reduction slightly elevated the 25(OH)D level by 1.5 ng/mL. They proposed that the release of vitamin D from fat and fat-free mass after weight loss was responsible for 25(OH)D elevation. Therefore, there is contradictory evidence regarding this issue, and the accurate relationship between vitamin D and adiposity indicators remains unclear.

According to the results of the studies included in the systematic review, overweight and obese individuals of different ages have similar chances of becoming vitamin D-deficient. Hence, age does not appear to have a significant impact on this association.

As a result of the heterogeneity in study characteristics, findings on vitamin D status and adiposity were inconsistent, with the inverse association being more prominent in females. It has been speculated that ethnicity, sex, and age may have a mediating effect on the relationship between 25(OH)D levels and anthropometric measures. It is likely that the differences in associations between females and males stem from the fact that women have a higher percentage of body fat and a different body composition than men. With the same BMI, men have less body fat than women. Thus, men store less vitamin D in adipose tissue and more remains in the blood. Furthermore, serum 25(OH)D levels are not stable throughout the year due to inadequate levels of 25(OH)D in the adipose tissue. In addition, vitamin D-binding protein could also contribute to sex differences in vitamin D status (94). It has been demonstrated that vitamin D-binding protein and adiposity are negatively correlated in men and positively correlated in women (94).

In reviewing the studies included in the systematic review, critical issues were raised, which could contribute to bias and confounding. There are several limitations, including heterogeneity in participant characteristics, the diversity of methods used to determine vitamin D levels and the analytical challenges involved, the use of variable definitions of hypovitaminosis D, the absence of adjustment for various confounding factors that influence vitamin D levels, and the reliability of various adiposity measures for describing obesity. The results of these studies may also be affected by a number of other factors related to the population studied, such as cultural and religious factors, dressing codes that mandate covering the majority of the body surface, and behavioral and lifestyle differences (95). It is also possible that variations in socioeconomic and developmental status could cause heterogeneity among studies that influence nutrition and lifestyle. Various socioeconomic and developmental factors influence the prevalence of obesity and vitamin D level (96, 97). Moreover, the amount of air pollution may affect vitamin D status, especially in urban areas where UVB wavelengths of sunlight are mostly blocked by pollutants (98). As a result of differences in health policies regarding the fortification of food with vitamin D in Europe and the USA and in national recommendations regarding vitamin D supplement use, vitamin D intake may differ significantly between countries (99). Vitamin D levels are also affected by genetic factors; however, without population-based genetic analyses, it is difficult to quantify their impact (100).

On the other hand, it has been suggested that low serum 25(OH)D concentrations reduce calcium absorption. PTH is secreted in response to low serum calcium concentrations, which stimulates the production of 1,25(OH)_2_D. The (nearly) normal serum levels of 1,25(OH)_2_D are maintained at the expense of high serum PTH concentrations, known as ‘secondary hyperparathyroidism’. Considering that serum 25(OH)D is the substrate for serum 1,25(OH)_2_D, serum 25(OH)D levels tend to decrease when serum 1,25(OH)_2_D increases. PTH may contribute to fat accumulation by increasing insulin resistance and inhibiting lipolysis (101). Additionally, vitamin D may regulate uncoupling proteins, which may play a role in energy metabolism (102). Despite this, Walsh *et al.* (53) found that PTH was not affected by BMI or sex and was not correlated with BMI. In obese individuals, there may be alterations in the relationship between 25(OH)D and PTH levels (53). To optimally determine the vitamin D status, the 25(OH)D threshold for maximum suppression of PTH has been suggested (103). A previous study showed that patients with a BMI ≥30 kg/m^2^ had a lower threshold for suppressing PTH levels (5 ng/mL) than patients with a BMI <30 kg/m^2^ (10 ng/mL) (104). This suggests that a very low 25(OH)D level is required to activate the PTH axis, leading to secondary hyperparathyroidism and bone loss (104).

The BMI has been used as an indicator of obesity in the majority of studies included in this systematic review. However, although BMI is the most widely accepted method for defining obesity, it is not an accurate measure of fat mass and distribution of body fat. Compared with subcutaneous fat, excess visceral fat confers a greater risk of metabolic and cardiovascular diseases for the same BMI value (105, 106). Several more meaningful measures of adiposity have been developed to resolve this methodological issue, including fat mass, WC, and WHR.

The present systematic review investigated the correlation between serum 25(OH)D and anthropometric and adiposity measurements in healthy individuals of various ages. However, some limitations must be considered. As not all included studies separately reported the relationship between serum vitamin D and adiposity measures in men and women, accurate estimates for men and women could not be provided. Different cutoff points were used for defining obesity and vitamin D deficiency in the included studies. This systematic review also examined the relationship between obesity and vitamin D deficiency using cross-sectional studies, which made the causality findings more difficult. Finally, we did not have access to the complete data of all related papers.

## Conclusion

Our systematic review highlights the prevalence of vitamin D deficiency among overweight/obese individuals and its inverse correlation with adiposity measures. Despite this association, it is essential to acknowledge the influence of various confounding factors, including dietary intake, physical activity, educational level, and seasonal variations, which could impact the serum 25(OH)D levels. Further prospective investigations are warranted to establish a causal relationship between vitamin D levels and obesity, shedding light on the underlying mechanisms. Additionally, the findings underscore the importance of monitoring serum vitamin D levels in individuals with excess weight. Considering the variability in climate and dietary patterns across different regions, standardizing the 25(OH)D cut-off points would benefit from additional research. Increased awareness of the interplay between obesity and vitamin D levels can prompt adjustments in clinical approaches among nutritionists and health-care professionals. Because the field of vitamin D research is dynamic and continues to evolve, and new studies may indeed provide further insights into the relationship between serum 25(OH)D levels and adiposity. In addition, the scarcity of cohort studies underscores the need for further longitudinal investigations to elucidate the causative mechanisms linking vitamin D with adiposity. So, we recommend that future researchers consider conducting updated systematic reviews to integrate the latest evidence on this important topic.

## Declaration of interest

The authors declare that there is no conflict of interest that could be perceived as prejudicing the impartiality of the study reported.

## Funding

This work did not receive any specific grant from any funding agency in the public, commercial, or not-for-profit sector.

## Data availability

The datasets used and/or analyzed during the current study are available from the corresponding author on reasonable request.

## Consent for publication

All authors have given consent for the paper to be published by the corresponding author.

## Author contribution statement

B.A. and F.H. designed and wrote the manuscript. S.H. and M.M. performed interpretation of results and critical revision of the manuscript. F.A. and M.V. critically revised the manuscript. All authors read and approved the final version.
